# Rigid Polyurethane Foams with Various Isocyanate Indices Based on Polyols from Rapeseed Oil and Waste PET

**DOI:** 10.3390/polym12040738

**Published:** 2020-03-26

**Authors:** Aiga Ivdre, Arnis Abolins, Irina Sevastyanova, Mikelis Kirpluks, Ugis Cabulis, Remo Merijs-Meri

**Affiliations:** 1 Latvian State Institute of Wood Chemistry, Polymer Laboratory, 27 Dzerbenes St., LV-1006 Riga, Latvia; arnisaabolins@gmail.com (A.A.); alexander.drachov@gmail.com (I.S.); mkirpluks@gmail.com (M.K.); cabulis@edi.lv (U.C.); 2 Riga Technical University, Institute of Polymer Materials, 3/7 Paula Valdena St., LV-1048 Riga, Latvia; Remo.Merijs-Meri@rtu.lv

**Keywords:** rigid polyurethane foams, bio-based polyols, renewable materials, thermal conductivity, dimensional stability, isocyanate index

## Abstract

Developing polyols derived from natural sources and recycling materials attracts great interest for use in replacing petroleum-based polyols in polyurethane production. In this study, rigid polyurethane (PUR) foams with various isocyanate indices were obtained from polyols based on rapeseed oil and polyethylene terephthalate (RO/PET). The various properties of the prepared PUR foams were investigated, and the effect of the isocyanate index was evaluated. The closed-cell content and water absorption were not impacted by the change of the isocyanate index. The most significant effect of increasing the isocyanate index was on the dimensional stability of the resulting foams. This is due to the increased crosslink density, as evidenced by the increased formation of isocyanurate and increase of the glass transition temperature. Additionally, the influence on compression strength, modulus, and long-term thermal conductivity were evaluated and compared with reference PUR foams from commercially available polyols. Rigid PUR foams from RO/PET polyol were found to be competitive with reference materials and could be used as thermal insulation material.

## 1. Introduction

Polyurethanes (PURs) have been widely used for different applications, such as coatings, adhesives, sealants, elastomers, resins, and foams. Most of these PUR materials are obtained from petrochemicals which are non-renewable, have low sustainability, and cause environmental concerns for society [1]. Due to that, as well as increasing emphasis on issues concerning waste disposal and depletion of non-renewable resources, the development and production of polyols derived from natural sources and recyclable materials attracts great interest [2].

Over the past few decades, oils and fats of vegetable origin have served as a viable alternative to petroleum resources [3]. Researchers have managed to obtain PUR materials with up to 20%–35% of renewable material content [4]. Polyols from vegetable oils, such as soybean [5,6], castor [7,8], palm oils [9,10], sunflower [11,12], and rapeseed oil (RO) [13,14] have been synthesized and investigated as alternatives to petrochemical polyols in the production of PURs. Rapeseed is an especially important oil plant in the temperate climate region. Its production is showing a growing trend, both in Europe and around the world [15,16]. 

Typical methods used to produce polyols from vegetable oils are applied for RO too: epoxidation and opening of oxirane rings [13,17], transesterification with triethanolamine, and transamidation with diethanolamine [17,18]. Hence, various RO polyols can be offered for the PUR market. According to Zieleniewska et al., investigations of PUR foams from RO polyols show competitive advantages such as lower water absorption (due to the hydrophobicity of fatty acids), higher thermal stability, and improved biological properties for rigid PUR foams. However, lower compressive strength and higher friability have been reported as disadvantages [13]. The incorporation of hard segments into the structure of PUR foam improves mechanical properties [19]. For this purpose, polyols from recycled polyethylene terephthalate (PET) can be successfully employed for PUR formulations to obtain rigid foams [20,21,22].

PET is one of the most versatile commodity thermoplastics, widely used in a broad range of applications, like fibres and disposable soft-drink bottles. The high popularity of PET as packaging material and its non-biodegradability creates huge amounts of waste. Therefore, effective recycling of PET is crucial [23,24]. PET waste can be recycled either physically or chemically.

One of the chemical recycling products of PET, that is suitable for obtaining PUR, is an aromatic polyester polyol. There are various chemical recycling pathways for aromatic polyester polyol synthesis: hydrolysis, aminolysis, methanolysis, and glycolysis [25]. Glycolysis is one of the most popular chemical recycling methods which has been commercialized [26]. Different depolymerization agents are employed (e.g., ethylene glycol [23,27], diethylene glycol (DEG) [28,29], propylene glycol [30], etc.) in glycolysis to obtain oligomers or oligoester diols/polyols with hydroxyl terminal groups. As mentioned before, aromatic polyester polyols improve the mechanical properties and thermal stability of PUR foams. Furthermore, it partly solves PET waste problems and decreases PUR dependence on petroleum feedstock. However, PET polyols’ high viscosity, fast crystallization, and incompatibility with a blowing agent were reported as its drawbacks [31], whereas polyols from plant oils do not have such problems. The combination of PET and natural oils, such as RO and tall oil, eliminates the mentioned disadvantages of aromatic polyester polyols. Novel polyols are compatible with a physical blowing agent, less viscous, and more stable against crystallization. Therefore, these polyols are suitable for the preparation of rigid PUR foams [21,32,33]. 

Rigid PUR and polyisocyanurate (PIR) foams are one of the most energy-efficient thermal insulation materials, which are mainly used in civil engineering as well as in the refrigeration industry. PUR and PIR foams are also applied as impact absorption materials for structural and functional engineering apllications [34]. PURs are made by the exothermic reactions between alcohols with two or more reactive hydroxyl groups (-OH) per molecule (diols, triols, polyols) and isocyanates that have more than one reactive isocyanate group (-NCO) per molecule (diisocyanates, polyisocyanates). Isocyanurate forms via trimerization of isocyanate as a side reaction during PUR formation [35].

The amount of PUR bonds and isocyanurate rings is closely related to a parameter called isocyanate index, which is the ratio of the equivalent amount of isocyanate used relative to the theoretical equivalent amount times 100. A theoretical equivalent amount is equal to one equivalent isocyanate per equivalent OH group. Higher isocyanate index incorporates more isocyanurate rings and increases the non-renewable material concentration in the end-product. The amount of isocyanurate rings in the polymer matrix affects the performance of PUR foams. The increase of glass transition temperature (T_g_), dimensional stability, thermal stability, as well as thermal insulation with increasing isocyanate index value is reported [17,36]. Formulations for the production of rigid PUR foams normally have an index between 105 and 125, and those for rigid PUR-PIR foams have an index between 180 and 350 [37]. Hence, there is no precise limit where PUR foams end and PIR foams start. The influence of isocyanate index on the performance of rigid PUR and PIR foams, based on polyols synthesised from both PET and natural oil, have not been previously reported.

In the present study, rigid PUR foams were obtained from polyols based on RO and PET. Different isocyanate indices (from 110 to 180) were used for development of PUR foam formulations. The various properties (apparent density, foaming parameters, dimensional stability, water absorption, glass transition temperature, compression strength, and thermal conductivity) of the prepared PUR foams were investigated. For comparison, three series of reference rigid PUR foams were obtained from commercially available polyols. The isocyanate index effect on the foams’ performance was evaluated, as well as optimal isocyanate index as a compromise of sustainable material content, and foam performance was determined.

## 2. Materials and Methods 

### 2.1. Materials

Aromatic polyester polyol based on RO and PET (RO/PET polyol, OH = 408 mg KOH/g) with RO/PET molar ratio 1:4 was synthesized, as described in our previous work [30]. Three base polyols for reference foams were chosen: aliphatic polyether polyol Lupranol 3300 (OH = 400 mg KOH/g, functionality f = 3) from BASF; aromatic polyester polyol Neopolyol 380 (OH = 370 mg KOH/g, f = 3.3 [4]) based on industrial PET waste and purchased from NEO Group (Lithuania); and RO/triethanolamine (RO/TEA) polyol (OH = 373 mg KOH/g, f = 2.6 [4]), which was synthesized by Latvian State Institute of Wood Chemistry via RO transesterification with TEA, and contains long dangling chains in its structure.

Rigid PUR foams were prepared by reacting synthesized RO/PET or reference polyols with different amounts of polymeric diphenylmethane diisocyanate (PMDI) (NCO group content = 31.5%) purchased from BASF. Other reagents used were: high functional polyether polyol Lupranol 3422 (OH = 490 mg KOH/g) from BASF, catalysts CAT NP-10 and 30 wt% solution of potassium acetate (KAc) in DEG from Performance Chemicals Handels, surfactant NIAX Silicone L6915 from Momentive Performance Materials, flame retardant Levagard PP (tris (2-chloroisopropyl)-phosphate)) from Lanxess, and physical blowing agent Solkane 365/227 (pentafluorobutane:heptafluoropropane = 87:13) from Solvay. These materials were used without any additional prior treatment. 

### 2.2. Preparation of Rigid PUR Foams

Rigid PUR foam series RO/PET was prepared from synthesized RO/PET polyol. In addition, three series of reference foams were prepared. As mentioned before, three base polyols for reference foams were chosen: Lupranol 3300, NEO 380, and RO/TEA. The obtained foam series were named LUPR, NEO, and RO/TEA, respectively. Chosen isocyanate indices were 110, 120, 130, 150, and 180. Foam samples were named SERIES NAME II = n, where n is the relevant isocyanate index. For example, RO/PET II = 120 is an RO/PET series rigid PUR foam with an isocyanate index of 120. 

Polyols’ systems were prepared by mixing all of its components according to the formulations presented in Table 1.

The necessary amount of PMDI was calculated according to the equation:

(1) $$m_{PMDI}=\frac{II}{w_{NCO}}\cdot \left(\sum^{\;}\frac{OH_{n}\cdot m_{n}}{1336}+4.67\cdot m_{H2O}\right)$$

where *II* is the isocyanate index; *m_PMDI_*, *m_n_*, and *m_H2O_* refer to the mass of PMDI, each polyol, and summary water, respectively; *w_NCO_* refers to the content of NCO groups of PMDI (31.5%); and *OH_n_* is the hydroxyl value of each polyol (mg KOH/g).

The calculated amount of PMDI was added to a polyol system, and the resulting mixture was stirred vigorously at 2000 rpm for 10 s. Afterward, it was instantaneously poured into an open mold with the dimensions 30 × 30 × 10 cm to obtain free-rise foams. After preparing all foam samples, they were allowed to cure at ambient conditions for 24 h before any further tests.

### 2.3. Characterization of Rigid PUR Foams

The process of rigid PUR foam formation was monitored by measuring foaming parameters: the duration of cream time, gel time, and tack-free time. The closed-cell content and apparent density were measured according to the standards ISO 4590 and ISO 845, respectively. The compression strength of rigid PUR foams were tested on testing machine Zwick/Roell Z100 (standard ISO 844, maximum load-cell capacity 1 kN, test speed—10%/min, six specimens for each composition). Cylinder specimens with diameter and height of ≈20 mm were cut with a drill press using a crown drill bit. Water absorption was tested immersing PUR specimens into the water for seven days, according to ISO 2896. The dimensional stability measurements were acquired according to ISO 2796. Two modes were chosen: 28 days at 70 °C, ambient relative humidity (R.H.); and 28 days at 70 °C, R.H. = 97%. The initial thermal conductivity of the rigid PUR foams was measured, as well as thermal conductivity after 24 weeks. These tests were carried out with Linseis Heat Flow Meter 200 according to the ISO 8301 standard. The temperature range was +10...+30 °C, and dimensions of specimens were 200 × 200 × 30 mm. The FTIR data was collected using an attenuated total reflectance technique with a ZnSe and Diamond crystals on a Thermo Fisher Nicolet iS50 spectrometer. A total of 32 scans were averaged at 4 cm^−1^ resolution for each spectrum. Dynamic mechanical analysis (DMA) was carried out with Mettler Toledo DMA/SDTA861^e^: temperature range from 25 to 200 °C, ramp rate of 3 °C/min, frequency of 1 Hz, amplitude 40 μm, and maximal force of 2 N. Compression oscillation mode was used. Three cylinder specimens with a diameter of ≈16 mm and a height of ≈8 mm were tested for each composition. Specimens were cut with a drill press using a crown drill bit. The maximum of the peak in the viscoelastic parameter tan δ was used to define T_g_.

## 3. Results and Discussion

### 3.1. Sustainable Material Content

**Figure 1:**
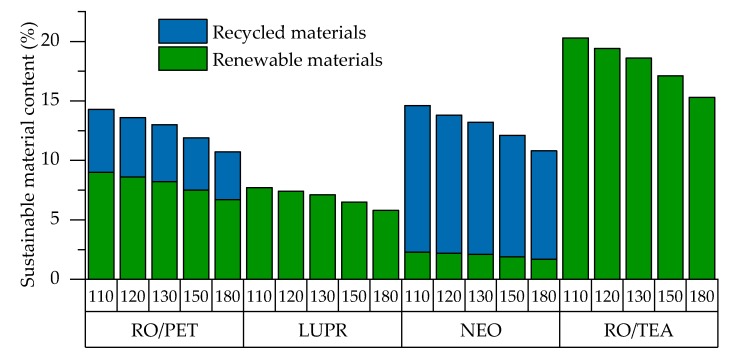
Sustainable material content in all series of rigid PUR foams.

Rigid PUR foams with different isocyanate indices were obtained by the free-rise method. Renewable and recycled material content was calculated and is shown in Figure 1. 

The amount of renewable and recycled materials in RO/PET foams is between 11% to 14%. In comparison, reference LUPR series foams contain up to 8%; NEO series, 15%; and RO/TEA series, 20%. The difference in sustainable material content between series of PUR foams depends mainly on the sustainability of the base polyol. The largest quantity of non-renewables in PUR formulations is made by PMDI. Hence, the sustainable material content decreases by rising isocyanate index. 

### 3.2. Apparent Density and Closed-Cell Content

The obtained rigid PUR foams were characterized by apparent density and closed-cell content (Table 2). Closed-cell content of RO/PET PUR foams was higher than 95 vol.% (on average, 97 vol.%) and was not impacted by the isocyanate index. Reference foams, LUPR (ρ = 40–43 kg/m^3^), NEO (ρ = 40–45 kg/m^3^), and RO/TEA (ρ = 41–48 kg/m^3^) series, showed slightly lower closed-cell content: 93 vol.%, 95 vol.%, and 93 vol.% on average, respectively. Closed-cell content 92–98 vol.% is suggested as a typical rigid PUR foam characteristic [38]. All obtained rigid PUR foams are suitable for thermal insulation according to this indicator.

### 3.3. Reactivity of Foam Formation

Reactivity of the polyol system is characterized by foaming parameters, such as cream time (the visible increase of foam volume), gel time (the transition from liquid to solid), and the tack-free time (when the outer surface of the foam is not sticky anymore). Gel time is the starting point of a stable network formation by intensive allophanate crosslinking as well as urethane and urea linkages [36]. Table 3 shows that all three foaming parameters increase with an increase in the isocyanate index. The absolute amount of foaming catalyst, PC CAT NP-10, was identical for all foams in the RO/PET series. Therefore, its part by weight to the total mass of foams decreased by increasing the isocyanate index, and that increased the cream time. The increasing tendency of gel time is due to the increased production of low molecular weight polymers, including the unreacted monomers, as the stoichiometric imbalance between the reacting groups is increased. Because of their low free energy, the low molecular weight species are typically exposed to the free surfaces, causing adhesiveness and prolonging tack-free time [36,39]. 

As mentioned earlier, the amount of foaming catalyst for reference foams was adjusted according to the reactivity of their polyol mixes. The series RO/TEA and RO/PET polyol systems were required to add less catalyst, as they showed higher reactivity. The higher reactivity was due to a tertiary amine group in the main polyol structure [32].

### 3.4. FTIR and DMA Results

It is expected that at a higher isocyanate index, the crosslinking density will increase. Two effects contribute to this. First, the functionality of PMDI is 2.7 and its molecular mass is relatively small (aprox. 381 g/mol), so it will work as a crosslinking reagent by itself. Second, when an excess of isocyanate is added, allophanates, biurets, and isocyanurates can be formed via side reactions. That will add additional crosslink points in the PUR polymer matrix, which gives a decrease in M_c_ and an increase in elasticity. Additionally, the increase in T_g_ is expected for higher crosslinking [40].

**Figure 2:**
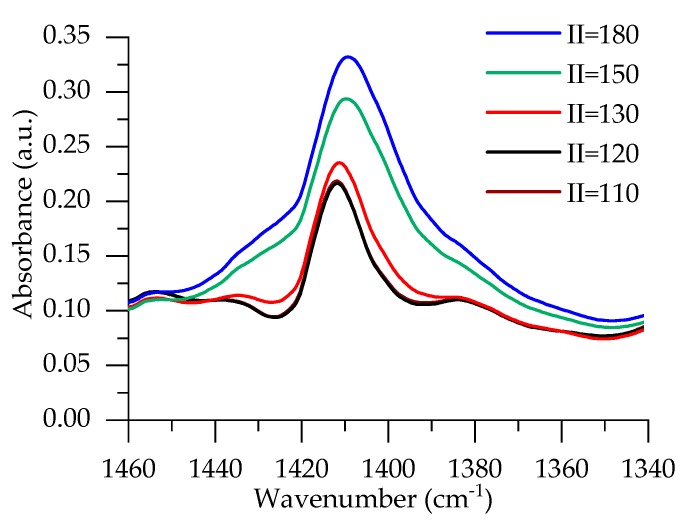
FTIR spectra for RO/PET rigid PUR foams, band at 1410 cm^−1^.

FTIR analysis was carried out for RO/PET series to judge the amount of isocyanurate formation. An isocyanurate band appears at 1410 cm^-1^, and it is shown in Figure 2. 

**Figure 3:**
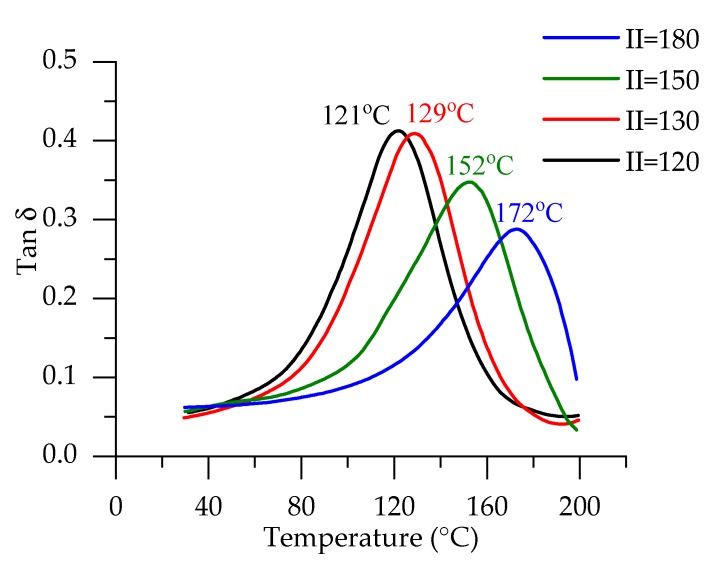
Damping factor curves of RO/PET series rigid PUR foams.

The peak intensity for foams with II = 110–130 is very similar. It is clearly seen that for higher isocyanate indices, more isocyanurates are formed. As no trimerization agent is added for formulations with II = 110–130, for those foams, crosslinking due to the formation of isocyanurate is closely similar. It agrees with the results of DMA. T_g_ was determined from damping factor curves, as shown in Figure 3. T_g_ of PUR foams for RO/PET II = 120 and II = 130 are similar: 121 °C and 129 °C, respectively. A higher increase of T_g_ is observed by increasing the isocyanate index, and it reached 172 °C for RO/PET II = 180. 

FTIR and DMA results prove that crosslink density increases because more crosslinks are formed due to the formation of isocyanurates. Additionally, an increase in elasticity can be expected [36].

### 3.5. Compression Strength

Compression strength is strongly affected by apparent density. Therefore, compression strength and modulus values for all samples were normalized for an apparent density of 45 kg/m^3^ using the equations of Hawkins et al [41]. Relating to the impact of isocyanate index, both decrease and increase in compression strength were reported. In the investigation of Javni et al., the soy polyol sample at the lowest index (II = 110) showed the highest compression strength because of the highest apparent density [42]. Additionally, Kim et al. showed a decrease of compression strength with an increase in isocyanate value (from 90 to 130) due to the decrease in apparent density [36]. 

**Figure 4:**
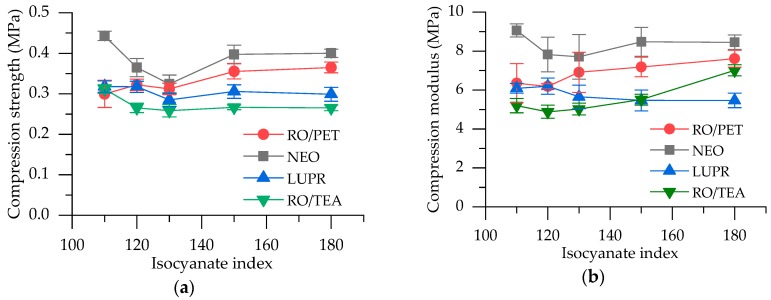
Physical-mechanical properties: (**a**) The compression strength of the RO/PET and reference rigid PUR foams; (**b**) Compression modulus of the RO/PET and reference rigid PUR foams.

In our case, the compression strength of RO/PET PUR foams slightly increases with an increase in isocyanate index (Figure 4a). It raises from 0.30 MPa for the sample with II = 110, to 0.37 MPa for the sample with II = 180. The same growing trend is observed for compression modulus, which raises from 6.35 MPa to 7.61 MPa (Figure 4b). 

Due to the enhancement of foam elasticity, anisotropic ratio (AR, strength ratio of parallel to perpendicular direction) for RO-PET 1/4 II = 180 is smaller (AR = 1.79) than for samples with lower isocyanate index (AR = 1.85–2.16). RO/PET shows higher compression strength than reference foams, LUPR and RO/TEA series, but slightly lower than the NEO series. NEO performance could be explained by the higher number of hard segments from recycled PET in its PUR foam matrix. 

Reference foams show an unexpected decrease in compression strength and modulus with an increase in isocyanate index from 110 to 130. It could be related to the lack of isocyanate trimerization catalyst in their formulations, hence no addition crosslinks are formed and unreacted isocyanate remains in foams. Nevertheless, all samples are within commercially acceptable limits. According to Javni et al., typical industrial PUR rigid insulating foams have compression strength at 10% strain between 0.15 and 0.25 MPa [42].

### 3.6. Dimensional Stability and Water Absorption

**Figure 5:**
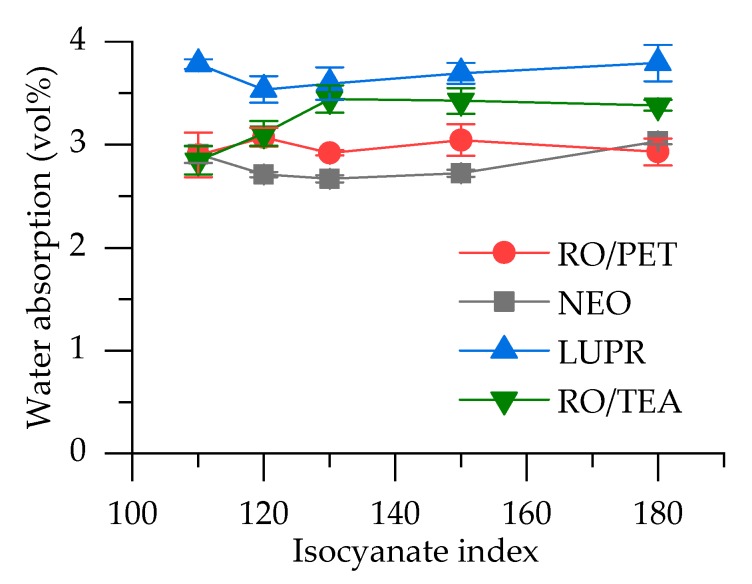
Water absorption after seven days of immersion.

The results of water absorption are presented in Figure 5. No impact of isocyanate index is observed for all series, with the exception of RO/TEA polyol based foam series, where a slight increase in water absorption for samples with II = 130, 150, and 180 is observed. PUR foams with base polyols containing aromatic structure (NEO and RO/PET series) show lower water absorption. 

**Figure 6:**
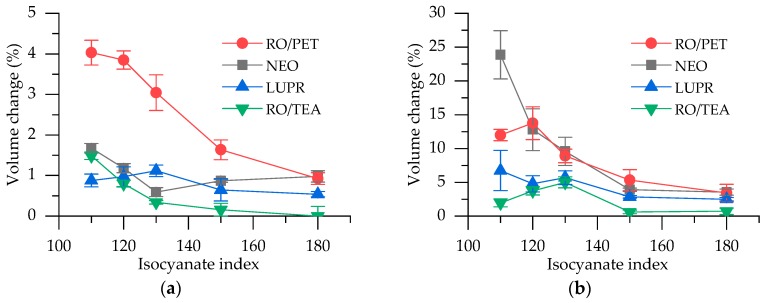
Dimensional stability–volume relative change at two modes: (**a**) 28 days at 70 °C, ambient relative humidity (R.H.); (**b**) 28 days at 70 °C, R.H. = 97%.

The increase of the isocyanate index improves dimensional stability due to the incorporation of the isocyanurate ring into the PUR foam matrix [43]. The results of dimensional stability are presented in Figure 6. Volume relative change after 28 days at 70 °C, ambient R.H., decreases from 4.0% to 1.0% with increasing isocyanate index. At mode where R.H. = 97%, volume change for RO/PET series is below 15% and reaches 3.4% when II = 180.

Although RO/PET polyols show better compatibility with blowing agents [32], which could improve dimensional stability, it was not observed in the results of the present study. As the standard ISO 2796 gives various options for modes and test duration, it is quite difficult to compare our results with other investigations. For example, Kim et al. suggested that volume change at 80 °C and −30 °C less than 1% in one day is desired for sufficient strength [36]. All of our series foams at 70 °C in one day showed volume change less than 1%, except for RO/TEA II = 110 (ΔV = 1.7%). Overall, starting from II = 130 and up, the dimensional stability is considered to be within commercially acceptable bounds. 

### 3.7. Thermal Conductivity

**Figure 7:**
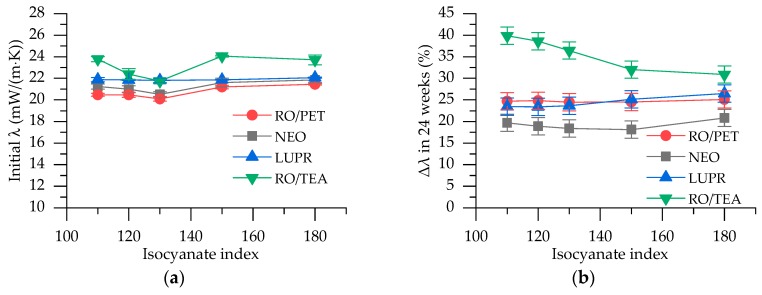
Results of thermal conductivity: (**a**) Initial thermal conductivity; (**b**) Thermal conductivity relative change in 24 weeks.

Initial thermal conductivity of rigid PUR foams with apparent density 42 ± 3 kg/m^3^ was measured, as well as thermal conductivity, after 24 weeks. The initial values are given in Figure 7a, where one can observe that foams from RO/PET polyol show as good thermal insulation property (average λ = 20.7 ± 0.7 mW/(m⋅K)) as foams from commercially used NEO (average λ = 21.2 ± 0.7 mW/(m⋅K)) and LUPR (average λ = 21.9 ± 0.2 mW/(m⋅K)). Rigid PUR foams from RO/TEA polyol showed slightly worse results for thermal conductivity (λ = 22.9 ± 1.2 mW/(m⋅K)). Additionally, it can be observed that the isocyanate index does not affect the initial thermal conductivity.

To evaluate the retention of thermal conductivity in the long term, Figure 7b is given with results of thermal conductivity relative change in 24 weeks. The best retention of thermal conductivity was shown in NEO series PUR foams from PET-based aromatic polyester polyol. The same results were observed in previous investigations by our research group [21,32]. The retention of thermal conductivity depends on the blowing agent gas diffusion rate through rigid PUR foam material. This is related to the crosslink density of PUR polymer matrix, cohesion energy, and ability of macromolecular conformation of PUR polymer chains. NEO and RO/PET polyol structure have aromatic groups derived from PET polymer and additional carboxylic groups that allow the formation of hydrogen bonds between polymer chains. Both of these aspects contribute to a stronger and more neatly packed polymer matrix, which minimizes the outward flow of the carbon dioxide (λ = 14.6 mW/(m⋅K)) and inward flow of air (λ = 24 mW/(m⋅K)), thus enhancing long-term thermal conductivity retention [44]. Galakhova et al. reported that carbon dioxide leaves rigid PUR foams already after 2.5 months [45].

No impact of isocyanate index on the retention of thermal conductivity for rigid PUR foams series NEO, RO/PET, and LUPR is observed. Only for PUR foams based on RO/TEA polyol, does the increase of isocyanate index improve retention. RO/TEA polyol chemical structure introduces long dangling chains into the PUR polymer matrix, which causes a low packing degree of PUR macromolecules [46]. The excess of PMDI introduces aromatic groups and increases crosslink density. As mentioned above, a more neatly packed polymer matrix is formed and long-term thermal conductivity retention is improved.

## 4. Conclusions

Rigid PUR foams with isocyanate index 110–180 were obtained from polyols based on rapeseed oil and polyethylene terephthalate. Additionally, reference PUR foams were obtained from commercially available polyols: Lupranol 3300, Neopolyol 380, and rapeseed oil/triethanolamine polyol. 

Closed-cell content of rapeseed oil/polyethylene terephthalate PUR foams was ≈97 vol.% and was not impacted by the isocyanate index. Additionally, no effect of the isocyanate index on water absorption was observed.

The values of foaming parameters increased with an increase in isocyanate index. The increasing tendency of gel time was due to the lower molecular weight polymers formed, which are typically exposed to the free surfaces, causing adhesiveness and prolonging tack-free time. 

The increase of compression strength, compression modulus, and dimensional stability of RO/PET PUR foams with the increase of isocyanate index were caused by the higher ratio of crosslinking due to the formation of isocyanurate (evidenced by FTIR and DMA).

Overall, the optimum complex of the physical and mechanical properties of RO/PET PUR foams was achieved for formulations with isocyanate indices of 130–180. Therefore, it is possible to obtain commercially competitive rigid PUR foams with sustainable material content up to 13%.

## Figures and Tables

**Table 1:** Formulations of rigid polyurethane (PUR) foams.

Component	Application	Amount, pbw ^1^
Rapeseed oil/PET (RO/PET) or reference polyol	Base polyol	75
Lupranol 3422	Crosslinking agent	25
Levagard PP	Flame retardant	20
NIAX Silicone L6915	Surfactant	2.0
30 wt% potassium acetate (KAc) solution in diethylene glycol	Catalyst	1.0–1.5 ^2^
PC CAT NP-10	Catalyst	1.6–4.0 ^3^
Water	Blowing agent	2.2
Solkane 365/227	Blowing agent	16

^1^ part by weight. ^2^ KAc was added only in PUR formulations with isocyanate indices 150 and 180. ^3^ 4.0 pphp were added for reference foams based on Lupranol 3300 to enhance reactivity.

**Table 2:** Apparent density and closed-cell content of RO/PET series rigid PUR foams.

Sample	Apparent Density, kg/m^3^	Closed-Cell Content, vol.%
RO/PET II = 110	39.1 ± 0.4	95.2 ± 0.2
RO/PET II = 120	38.5 ± 0.1	98.0 ± 0.4
RO/PET II = 130	44.3 ± 1.3	97.6 ± 0.3
RO/PET II = 150	39.1 ± 0.4	98.7 ± 0.2
RO/PET II = 180	41.7 ± 0.1	97.4 ± 0.4

**Table 3:** Foaming parameters.

	Time, s		
Sample	Cream	Gel	Tack-Free
RO/PET II = 110	22	52	75
RO/PET II = 120	22	52	75
RO/PET II = 130	23	54	90
RO/PET II = 150	25	54	93
RO/PET II = 180	25	56	108
